# Role of transcription regulatory sequence in regulation of gene expression and replication of porcine reproductive and respiratory syndrome virus

**DOI:** 10.1186/s13567-017-0445-2

**Published:** 2017-08-10

**Authors:** Chengbao Wang, Han Meng, Yujin Gao, Hui Gao, Kangkang Guo, Fernando Almazan, Isabel Sola, Luis Enjuanes, Yanming Zhang, Levon Abrahamyan

**Affiliations:** 1 Department of Preventive Veterinary Medicine, College of Veterinary Medicine, Northwest A&F University, No. 22 Xinong Road, Yangling, Shaanxi, 712100 China; 2 Swine and Poultry Infectious Diseases Research Center (CRIPA) and Research Group on Infectious Diseases in Production Animals (GREMIP), Faculty of Veterinary Medicine, Université de Montréal, 3200 Sicotte, Saint-Hyacinthe, QC J2S 2M2 Canada; 3 Department of Molecular and Cell Biology, Spanish National Centre for Biotechnology, (CNB-CSIC), C/Darwin No. 3, Campus Universidad Autonoma. Cantoblanco, 28049 Madrid, Spain; 4 Faculty of Veterinary Medicine, Department of Pathology and Microbiology, Université de Montréal, 3200 Sicotte, Saint-Hyacinthe, QC J2S 2M2 Canada

## Abstract

In order to gain insight into the role of the transcription regulatory sequences (TRSs) in the regulation of gene expression and replication of porcine reproductive and respiratory syndrome virus (PRRSV), the enhanced green fluorescent protein (EGFP) gene, under the control of the different structural gene TRSs, was inserted between the N gene and 3′-UTR of the PRRSV genome and EGFP expression was analyzed for each TRS. TRSs of all the studied structural genes of PRRSV positively modulated EGFP expression at different levels. Among the TRSs analyzed, those of GP2, GP5, M, and N genes highly enhanced EGFP expression without altering replication of PRRSV. These data indicated that structural gene TRSs could be an extremely useful tool for foreign gene expression using PRRSV as a vector.

**Electronic supplementary material:**

The online version of this article (doi:10.1186/s13567-017-0445-2) contains supplementary material, which is available to authorized users.

## Introduction, methods, and results

Porcine reproductive and respiratory syndrome virus (PRRSV) is the causative agent of porcine reproductive and respiratory syndrome (PRRS), which leads to a highly contagious respiratory disease in nursery pigs and reproductive failure in sows [1]. PRRS was first reported in 1987 in North America and has become pandemic within a few years. PRRS is still one of the most economically important diseases for the swine industry worldwide [2, 3]. PRRSV is a member of the genus *Arterivius* of the family *Arteriviridae* within the order *Nidovirales*. This enveloped virus bears a single-stranded, positive-sense RNA genome containing at least seven genes, encoding the replicase (ORF 1a and ORF 1b) and the structural proteins E, GP2 or GP2a, GP3, GP4, 5a, GP5, M, and N in the order 5′-ORF1-E-GP2-GP3-GP4-5a-GP5-M-N-3′ [4–6]. The structural proteins are expressed by a nested series of subgenomic (sg) RNAs, which are produced during viral transcription. The structure of the arterivirus and coronavirus sg mRNAs derives from the discontinuous step of minus-strand RNA synthesis, which is guided by conserved AU-rich transcription-regulating sequences (TRS) [7–9]. There are two key elements of TRSs that are present both at the 3′ end of the leader sequence (leader TRS) and at the 5′ end of each gene in the 3′-proximal region of the genome (body TRSs). The body TRS motifs are found preceding almost all structural genes, while a leader TRS is present at the 5′ end of the genome. Minus-strand RNA synthesis is guided by base-pairing between the genomic leader TRS and the copy of the body TRS present in the 3′ end of the nascent minus strand. Next, the nascent strands are extended with the complement of the genomic leader sequence, generating a nested set of minus-strand templates that can be directly copied into the sg mRNAs [10, 11]. Leader TRS is highly conserved among *Arteriviridae*. For example, in equine arteritis virus (EAV) the TRS contains the conserved hexanucleotide sequence UCAACU [12], highly related with those found in lactate dehydrogenase-elevating virus (LDV) (UAUAACC) [13] and in simian hemorrhagic fever virus (SHFV) (UUAACC) [14]. In case of PRRSV, the leader TRS is also highly conserved and appears to be UUAACC regardless of the PRRSV genotype (type 1 or type 2, also called European and North American genotypes, respectively) [15, 16]. In contrast, different body TRSs have been shown to be diverse: [U/A/G][U/A/G][A/C][A/G][C/U]C among North American genotype viruses, and U[A/U/C][A/G][A/C]CC among European genotype viruses. Furthermore, the number of body TRSs and sites upstream of the start codon of each ORF vary in length and sequence [16–18]. Others and we have previously shown that the infectious clone of PRRSV can be engineered as an expression vector, in which a foreign gene could be expressed under the control of a body TRS as a separate transcription unit. These findings have confirmed the potential use of PRRSV as a vaccine vector against swine pathogens [19–23].

**Figure 1:**
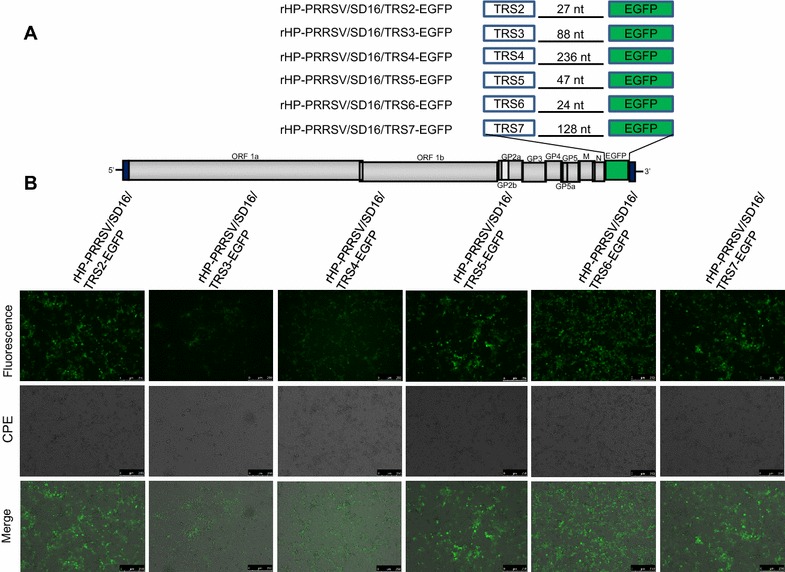
**Schematic diagram of rHP-PRRSVs containing EGFP gene under the control of TRSs and detection of EGFP expression by fluorescence microscopy. A** EGFP gene was under the control of the different structural genes TRSs, located upstream of the start codon of each structural genes and varied in length and sequence. Each transcriptional unit containing the EGFP gene and six different length TRSs of HP-PRRSV was inserted between the N protein and 3′-UTR in the HP-PRRSV genome. **B** Marc-145 cells infected with six different recombinant HP-PRRSVs expressing the EGFP gene were observed for CPE and fluorescence detection. Live cells were analysed by phase contrast and fluorescence microscopy.

**Figure 2:**
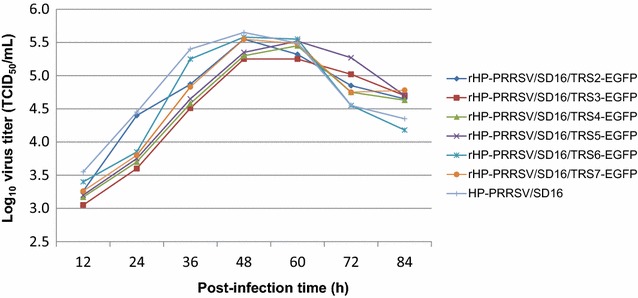
**In vitro replication of six rHP-PRRSVs containing EGFP gene in Marc-145 cells.** In vitro replication of the recombinant HP-PRRSVs was evaluated in Marc-145 cells infected at an MOI of 0.01. The viral titres were determined by using the Reed–Muench method.

The body TRS, including the conserved hexanucleotide motif and poorly conserved flanking sequences, form secondary structures essential for the sgRNA formation and play an important role in the regulation of viral transcription and translation [8, 24]. It was shown that a recombinant PRRSV vector with green fluorescent protein (GFP) gene driven by the body TRS2 and with an additional synthetic TRS6 controlling the ORFs 2a and 2b was stably able to express the foreign GFP gene even after 37 serial passages [19]. In addition, several studies have indicated that the presence of overlapping genes in EAV and PRRSV genomes represents a major challenge for the mutational analysis of the N- and C-termini of the structural proteins and also make it difficult to insert heterologous genes into the viral genome [25, 26]. Similarly, the presence of overlapping genes in the PRRSV genome is a serious obstacle to determining the role of other body TRSs in PRRSV gene expression. Based on this information, we hypothesized that different PRRSV body TRSs would lead to differential expression of a foreign gene (for TRS sequences information, please see Additional file 1). By using a reverse genetics system, we have evaluated the individual role of body TRSs (of type 2 genotype) of each of the six PRRSV structural genes in expression of a foreign gene. We rescued a series of recombinant PRRSVs expressing enhanced GFP (EGFP) driven by the six different body TRSs that corresponds to each PRRSV structural gene (Figure 1). Each transcriptional unit, including the individual body TRS and EGFP gene, was inserted between the N protein and 3′-UTR in a full-length cDNA infectious clone of HP-PRRSV/SD16 strain (Figure 1A). This position has proven to express foreign genes stably without affecting PRRSV replication [19–23]. The six recombinant HP-PRRSVs were recovered as previously described [20] and the EGFP expression was analyzed in virus-infected Marc-145 cells using fluorescent microscopy (Figure 1B). Different patterns of EGFP expression were observed. The TRSs of GP2, GP5, M, and N genes exhibited a relatively greater ability to control EGFP expression compared with the TRSs of GP3 and GP4 (Figure 1B). In order to investigate whether the rescue procedure or exogenous gene insertion affected the replication ability of the recombinant viruses, the growth characteristics of the six recombinant HP-PRRSVs were evaluated in a time-course experiment. The replication patterns of the recombinant HP-PRRSVs were compared with those of the parental virus by examining the growth kinetics in Marc-145 cells infected with a multiplicity of infection (MOI) of 0.01 PFU/cell [20]. Our results demonstrated the similar patterns in growth rate and maximum titers for the parental virus and all the recombinant HP-PRRSVs containing the individual body TRS and EGFP gene inserted between the N protein and 3′-UTR (Figure 2), indicating that the addition of different body TRSs in the EGFP transcriptional unit did not affect viral replication.

**Figure 3:**
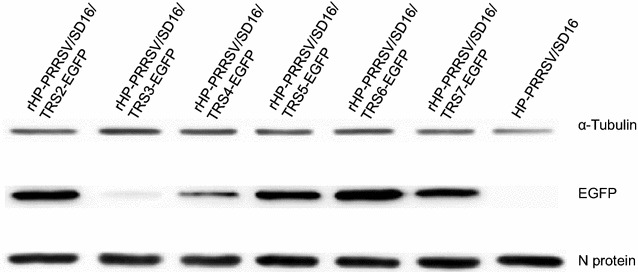
**Effect of the HP-PRRSV TRSs on EGFP expression in Marc-145 cells infected with rHP-PRRSVs.** Marc-145 cells were infected at an MOI of 0.01 and cultured until 60% of cells showed the cytopathic effect (CPE). For western blot analysis of EGFP production, total proteins were collected from virus-infected cells, electrophoresed, transferred to a polyvinylidene difluoride (PVDF) membrane, and immunostained using a mouse anti-GFP monoclonal antibody, anti-α-Tubulin antibody and anti-PRRSV N protein as a loading control.

**Figure 4:**
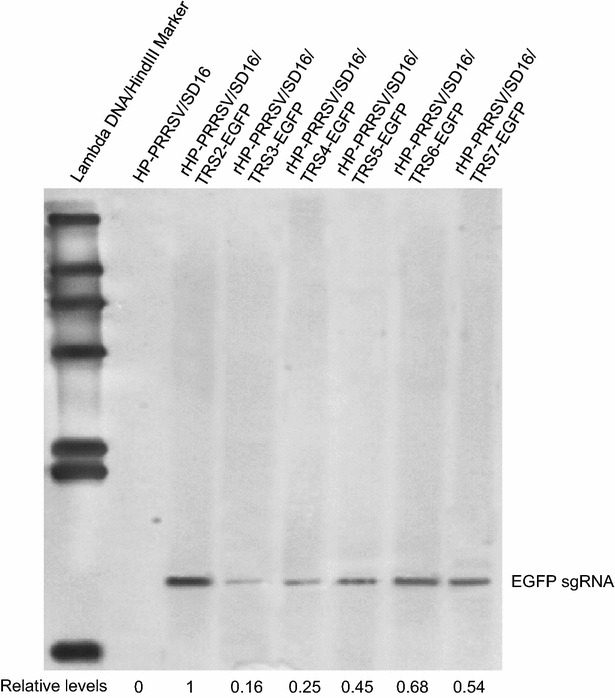
**Detection of EGFP mRNAs of the recombinant HP-PRRSVs using northern blot analysis.** Total RNAs from Marc-145 cells infected with six different recombinant HP-PRRSVs expressing EGFP and the parent strain were separated on a Tris–borate–EDTA–urea-15% polyacrylamide gel. The gel was transferred onto a piece of membrane (Hybond N+; Amersham). The blot was UV cross-linked using a cross-linking system (HL-200 HybriLinker; UVP), and DIG-labelled oligonucleotides were used as probes for EGFP sgRNA detection. The sequences for the probes used were as follows: SNB041-F, 5′-GTGAGCAAGGGCGAGGAG-3′; and SNB041-R, 5′-GTAGTGGTTGTCGGGCAGCA-3′. Numbers below the northern bands indicate relative levels of EGFP sgRNA of each recombinant virus compared to EGFP sgRNA of rHP-PRRSV/SD16/TRS2-EGFP. ImageJ software (NIH) was used to quantify the signal from the gel.

In order to gain insight into the different effect of the six body TRSs of HP-PRRSV on regulation of EGFP gene expression, the EGFP production in virus-infected Marc-145 cells was analyzed by western blot analysis. Different levels of EGFP production by the six rescued viruses containing EGFP transcriptional units between the N protein and 3′-UTR were observed. Among them, rHP-PRRSV/SD16/TRS2-EGFP, rHP-PRRSV/SD16/TRS5-EGFP, rHP-PRRSV/SD16/TRS6-EGFP and rHP-PRRSV/SD16/TRS7-EGFP produced higher levels of EGFP than other investigated recombinant viruses (Figure 3). In addition, quantitative comparison of EGFP expression levels in virus-infected Marc-145 cells were also analyzed by using flow cytometry (FACS Aria II; BD Bioscience) and a GFP Quantification Kit (BioVision, Mountain View, CA, USA) as previously described (data not shown) [20]. Overall, the fluorescent intensity measured was almost associated with the levels of by the western blot and EGFP fluorescence levels observed in virus-infected cells. On the other hand, insertion of an additional transcriptional unit into the virus genome might affect the efficient incorporation of structural proteins into virions [27–29]. In order to evaluate the potential effect of the six body TRSs on the expression of the viral structural proteins, the production of the N protein was also analyzed in virus-infected Marc-145 cells by western blot analysis (Figure 3). No significant differences in N expression were detected in cells infected with the different recombinant viruses and the parental virus. The results presented here (Figure 3) and our earlier findings had shown that insertion of the EGFP transcriptional units between N gene and 3′-UTR did not affect production of viral structural proteins [23–25]. Furthermore, we determined EGFP mRNA levels in virus-infected Marc-145 cells by Northern blot analysis (Figure 4). These experiments should gain insight into the different effect of the six body TRSs of HP-PRRSV on the regulation of EGFP gene transcription. Taken together, a comparison of EGFP transcription levels between the recombinant EGFP viruses and the parental virus demonstrated that body TRSs of GP2, GP5, M and N genes showed higher levels of EGFP expression than TRSs of GP3 and GP4 without altering the HP-PRRSV replication.

## Discussion

Previous studies and our own findings have suggested that the body TRS2 and TRS6 of PRRSV can play important roles in the regulation of viral transcription and translation [19–23]. However, there have been no studies, as far as we know, that have addressed the roles of the other body TRSs in gene expression regulation, replication, and transcription of PRRSV because the overlapping genes in PRRSV genome made mutation analysis challenging. It is well-accepted that leader TRS and body TRSs are the two key elements of PRRSV transcription. However, the roles and efficiency of base-pairing interaction between the leader TRS and different body TRSs on the expression of different structural proteins are not discussed in this paper due to the space limitation. Nonetheless, whether the distance between TRS and downstream gene governs the efficiency of the transcription and (possibly) translation is the objective of our further research. Therefore, we report here, for the first time, only the data that allow comparison of the effects of body TRSs on the expression of a foreign gene. We generated a series of recombinant HP-PRRSVs expressing EGFP gene driven by the six individual body TRSs of each HP-PRRSV structural genes, respectively. Each transcriptional unit, including the individual body TRS and EGFP gene, was inserted between the N protein and 3′-UTR in a full-length cDNA infectious clone of HP-PRRSV/SD16 strain. Importantly, all six recombinant HP-PRRSVs showed similar patterns of growth rate and maximum titers in comparison with the parental virus. These data indicated that the site between N gene and 3′-UTR can tolerate the addition of a foreign gene without reduction of the level of the viral replication.

Insertion of an additional transcriptional unit into the virus genome might affect the efficient incorporation of structural proteins into virions [27–29]. In this study, six recombinant HP-PRRSVs were subjected to western blot by measuring the ratios of the N protein and EGFP protein, respectively. The present results and our earlier data showed that insertion of the EGFP transcriptional units between N gene and 3′-UTR did not affect the incorporation of viral protein into the virions by measuring the ratios of the N protein and EGFP protein [20–22]. Moreover, six recombinant HP-PRRSVs were subjected to Northern blot analysis by measuring the ratios of the EGFP mRNA. Overall, the body TRSs of GP2, GP5, M and N genes produced the higher level of EGFP expression when this reporter gene is cloned upstream of the N gene and 3′-UTR, suggesting that these body TRSs at this position would assure effective regulation of the gene of interest. It is possible that HP-PRRSV has evolved to have unique body TRSs for each structural gene, and they are most effective in regulating the expression of the corresponding structural genes at their original positions. In summary, we have evaluated the role of six PRRSV body TRSs in expression of a foreign gene by using HP-PRRSV reverse genetics system. We showed that HP-PRRSV body TRSs have the ability to regulate gene expression, replication, and transcription of the foreign gene at different levels. Moreover, our results and the previous findings all indicate that the PRRSV body TRSs could be a useful tool for controlling foreign gene expression. Compared with the expression levels of six different recombinant PRRSVs expressing EGFP gene, body TRSs of GP2, GP5, M and N genes have shown relatively higher levels of EGFP expression without altering the viral replication. Therefore, our results provide new clues useful for the rational design of next generation effective PRRSV vaccine vectors.

## Additional file

**Additional file 1.** **The TRS sequences of structural genes in the HP-PRRSV/SD16 genome (GenBank: JX087437).**
