# Erratum to: Role of transcription regulatory sequence in regulation of gene expression and replication of porcine reproductive and respiratory syndrome virus

**DOI:** 10.1186/s13567-017-0464-z

**Published:** 2017-09-20

**Authors:** Chengbao Wang, Han Meng, Yujin Gao, Hui Gao, Kangkang Guo, Fernando Almazan, Isabel Sola, Luis Enjuanes, Yanming Zhang, Levon Abrahamyan

**Affiliations:** 1 Department of Preventive Veterinary Medicine, College of Veterinary Medicine, Northwest A&F University, No. 22 Xinong Road, Yangling, Shaanxi 712100 China; 2 Swine and Poultry Infectious Diseases Research Center (CRIPA) and Research Group on Infectious Diseases in Production Animals (GREMIP), Faculty of Veterinary Medicine, Université de Montréal, 3200 Sicotte, Saint-Hyacinthe, QC J2S 2M2 Canada; 3 Department of Molecular and Cell Biology, Spanish National Centre for Biotechnology, CNB-CSIC, C/Darwin No. 3, Campus Universidad Autonoma. Cantoblanco, 28049 Madrid, Spain; 4 Faculty of Veterinary Medicine, Department of Pathology and Microbiology, Université de Montréal, 3200 Sicotte, Saint-Hyacinthe, QC J2S 2M2 Canada

## Erratum to: Vet Res (2017) 48:41 DOI 10.1186/s13567-017-0445-2

After publication of the article [1], it has been brought to our attention that an acknowledgement has been omitted from the original article. The authors would like to include the following, The authors also thank Prof. En-Min Zhou (Northwest A&F University) and his laboratory for technical support.”

## References

[1] Wang C, Gao Y, Gao H, Guo K, Almazan F, Sola I, Enjuanes L, Zhang Y, Abrahamyan L (2017). Role of transcription regulatory sequence in regulation of gene expression and replication of porcine reproductive and respiratory syndrome virus. Vet Res..

