# Supplementary material for: Role of transcription regulatory sequence in regulation of gene expression and replication of porcine reproductive and respiratory syndrome virus
Source: Vet Res. 2017 Aug 10;48:41. doi: 10.1186/s13567-017-0445-2 (PMC5553793; doi:10.1186/s13567-017-0445-2)
Supplement: Supplementary file 1 — Additional file 1. The TRS sequences of structural genes in the HP-PRRSV/SD16 genome (GenBank: JX087437). [file 13567_2017_445_MOESM1_ESM.doc]

**Additional files**

**Supplemental table: The TRS sequences of structural genes** in the HP-PRRSV/SD16 genome (GenBank: JX087437)

| The name of structural gene TRS | The sequence of body TRS used | The distances between TRSs and their initial sites of corresponding genes* |
| --- | --- | --- |
| TRS2 | TTGAACCA | 27 |
| TRS3 | TAACCAT | 88 |
| TRS4 | TTGACCA | 236 |
| TRS5 | ATTAGCCTGTC | 47 |
| TRS6 | TTTAACCA | 24 |
| TRS7 | TAACCA | 128 |

*This refers to the number of nucleotides between the first nucleotide of the TRSs and the first letter of the start codon of the corresponding ORFs.
